# Functional Correlates of Action Observation of Gait in Patients with Parkinson's Disease

**DOI:** 10.1155/2020/8869201

**Published:** 2020-12-29

**Authors:** Giulia Bommarito, Martina Putzolu, Laura Avanzino, Carola Cosentino, Alessandro Botta, Roberta Marchese, Matilde Inglese, Elisa Pelosin

**Affiliations:** ^1^Department of Neuroscience, Rehabilitation, Ophthalmology, Genetics and Maternal Child Health, University of Genova, Genova, Italy; ^2^Department of Experimental Medicine, Section of Human Physiology, University of Genova, Genova, Italy; ^3^IRCCS Ospedale Policlinico San Martino, Genova, Italy

## Abstract

**Background:**

Action observation (AO) relies on the mirror neuron system (MNS) and has been proposed as a rehabilitation tool in Parkinson's disease (PD), in particular for gait disorder such as freezing of gait (FOG). In this study, we aimed to explore the brain functional correlates of the observation of human gait in PD patients with (FOG+) and without (FOG-) FOG and to investigate a possible relationship between AO-induced brain activation and gait performance.

**Methods:**

Fifty-four participants were enrolled in the study (15 PD FOG+; 18 PD FOG-; 21 healthy subjects (HS)) which consisted of two tasks in two separate days: (i) gait assessment and (ii) task-fMRI during AO of gait. Differences between patients with PD (FOG+ and FOG-) and HS were assessed at the level of behavioral and functional analysis.

**Results:**

Gait parameters, including gait velocity, stride length, and their coefficients of variability (CV), were different in PD patients compared to HS, whereas gait performance was similar between FOG+ and FOG-. The PD group, compared to HS, presented reduced functional activation in the frontal, cingulum, and parietooccipital regions. Reduced activity was more pronounced in the FOG+ group, compared to both HS and FOG- groups. Gait variability positively correlated with precuneus neural activity in the FOG+ group. *Discussion*. Patients with PD present a reduced functional activity during AO of gait, especially if FOG+. A baseline knowledge of the neural correlates of AO of gait in the clinical routine “on” status would help for the design of future AO rehabilitative interventions.

## 1. Introduction

The observation of someone performing an action recruits brain area that is activated also during the action execution. The physiological bases of this phenomenon rely on the mirror neuron system (MNS) [1, 2]. The MNS has been identified as the neural substrate for action observation (AO) training, observation plus repetition of actions, which has been proposed as a rehabilitation strategy in neurological disorders, including Parkinson's disease (PD) [3]. However, whether the MNS, and thus the efficacy of AO, is preserved or altered in patients with PD is still controversial [4]. Indeed, voluntary movement imitation seems to be preserved [5] and movement observation is accompanied by bilateral beta reduction in subthalamic power and cortico-subthalamic coherence [6]. On the contrary, the modulation of motor-evoked potentials [7] as well as the event-related mu rhythm desynchronization [8] during AO has been showed to be impaired in PD.

Gait disorders are frequent in PD patients, and among them, freezing of gait (FOG) affects up to 54% of patients [8]. However, in spite of their major effects on disability, their neural correlates remain quite unknown. Most of the studies exploring the neural correlates of FOG in PD have been performed with fMRI by a motor imagery task, revealing the involvement of the mesencephalic locomotor regions, basal ganglia, and supplementary motor and parietal cortices [9, 10]. The activation in the premotor, parietal, and pontomesencephalic regions in patients with FOG (FOG+) was modulated by the antiparkinsonian treatment [11].

It has been demonstrated that AO training is effective in improving gait disturbances such as FOG [12]. Agosta and coworkers showed that PD patients with FOG exhibited a clinical improvement and increased recruitment of cortical areas involved in motor and attentional control, after a training based on AO [13]. However, in this study, only PD patients with FOG were included, while data in the literature about the neural correlates of AO of gait comparing PD patients with and without FOG are still missing. In addition, it should be considered that most previous studies have explored brain activity during gait in PD patients in the “off” state [14–16]. Although this may unveil the functional reorganization and reflect more accurately the pathophysiological substrate of the disease, it prevents the comprehension of the neural functional mechanisms underlying behavioral performance in the everyday life of PD patients. Thus, a better understanding of the functional changes occurring during AO of gait in patients with or without FOG under dopaminergic treatment would help lay the grounds for the design of more effective rehabilitative strategies for PD. This prompted us to design a functional MRI (fMRI) study (i) to explore the neural structures recruited during the observation of human gait in PD patients and (ii) to detect possible differences between PD patients with (FOG+) and without (FOG-) FOG while in the “on” state. Further, we aim also (iii) to assess whether there is a relationship between AO-induced brain activation and gait performance.

## 2. Methods

### 2.1. Subjects

A total of 54 subjects, 33 PD subjects and 21 healthy subjects (HS), were prospectively enrolled in the study. Participants with PD were recruited at the Department of Neuroscience, University of Genova. Healthy subjects were recruited from a local community as the control group. The study was approved by the regional ethical committee, and written informed consent was obtained from each participant prior to study entry. Common inclusion criteria were as follows: (i) >25/30 at the Mini-Mental State Examination (MMSE) and (ii) able to walk for 5 minutes unassisted. PD patients were included if they had idiopathic PD, as defined by the UK Brain Bank criteria, were in Hoehn and Yahr stage II–III, and were medically stable for at least 3 months prior to the study. Exclusion criteria, based on patients' report and medical records, for all participants included the following: (i) history of neurologic disorders other than PD, (ii) psychiatric comorbidity (e.g., major depressive disorder as determined by DSM IV criteria), (iii) contraindications to magnetic resonance imaging (MRI) exam, and (iv) visual impairments that could hinder task functional MRI (task-fMRI) acquisition.

### 2.2. Study Design and Procedures

The study consisted of two separate experimental sessions (gait evaluation and AO task-fMRI) performed by each participant in two different days. On the first day, demographic and clinical characteristics were collected, and then, participants were randomly assigned to either gait assessment evaluation first or AO task-fMRI first by a computerized block randomization, with a block size of 6. On the second day (≈after 7 days), subjects performed the other part of the study protocol. All PD patients were under treatment with dopaminergic therapy, and evaluations took place during the “on” state (≈1 hour after taking antiparkinsonian medications).

### 2.3. Demographics and Clinical Evaluations

Age, sex, and education were recorded for each participant along with other subject characteristics. For PD participants, disease severity was evaluated with section III of the MDS-Unified Parkinson Disease Rating Scale [17]. The Montreal Cognitive Assessment (MoCA) was used to evaluate global cognitive dysfunction [18]. The new FOG questionnaire [19] and the rapid 360-degree turn in place task [20] were used for evaluating the presence and the severity of FOG.

### 2.4. Action Observation: Task-fMRI

The MRI protocol was aimed at assessing the functional activity during AO of gait; we chose a block fMRI design with 30 seconds of rest (one block) followed by 30 seconds of task, 8 blocks total. Subjects were required to watch a third-person video clip representing human walking, inside the magnetic resonance scanner. Participants watched the video clip by looking at the mirror positioned on the head coil. The mirror reflected the human gait video displayed on a screen placed inside the magnet room, located ≈1 m far from the bottom of the scan. A custom-made Matlab® software synchronized video clip onset with fMRI acquisition.

### 2.5. Functional MRI Image Acquisition and Preprocessing

Images were acquired on Signa Excite 1.5 MRI (Signa Excite General Electric Healthcare, USA) with an 8-channel phased-array head coil. The MRI protocol included a T2-weighted sequence (TR/TE = 2340/102 ms, voxel size: 0.94 × 0.94 × 4 mm^3^), Fast Spoiled Gradient Echo (FSPGR) 3D T1-weighted sequence (TR/TE = 11.70/5.12, voxel size: 1 × 1 × 1 mm^3^), and a single-shot echo-planar imaging (EPI) sequence (TR/TE = 3000/60 ms, slice thickness = 4 mm, pixel size = 3.75 mm^2^) for task-fMRI during action observation of gait.

The initial preprocessing step included the despiking (detection and reduction of extreme time series outliers by fitting a smooth curve insensitive to extreme outliers to the data), performed in AFNI (https://afni.nimh.nih.gov) [21]. Brain extraction was performed with FreeSurfer skull stripping on the T1-weighted anatomical sequence. The other preprocessing steps were performed using FSL [22] (FMRIB's Software Library, https://fsl.fmrib.ox.ac.uk/fsl/fslwiki) as implemented in FEAT [23], including removal of the first 3 volumes, motion correction using MCFLIRT (https://fsl.fmrib.ox.ac.uk/fsl/fslwiki/MCFLIRT) [24], slice timing correction for regular ascending acquisition (using Fourier-space time series phase shifting), spatial smoothing (Gaussian kernel, full width at half maximum of 6 mm), grand-mean intensity normalization of all volumes by a single multiplicative factor, and high-pass temporal filtering (Gaussian-weighted least-squares straight-line fitting, sigma = 30 sec). T1-weighted brain images were segmented into white matter (WM), grey matter (GM), and cerebrospinal fluid (CSF) using FAST; then, the WM and CSF masks were registered to the functional space and the average of the raw time series within each mask was derived in order to obtain the nuisance signal from WM and CSF. Boundary-based registration BBR [25] was used to register each individual functional data to the corresponding T1-weighted brain image. Then, linear affine 12-degree of freedom registration was performed to register each subject's T1-weighted brain to the standard space (MNI152 brain template, voxel size: 2 mm^3^ [21].

### 2.6. Gait Evaluation

**Figure 1:**
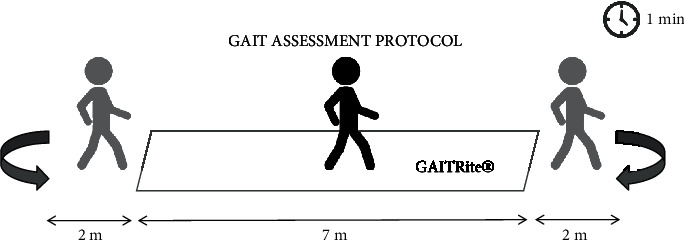
Gait assessment protocol. PD participants walk at their comfortable speed on GAITRite® for 1 minute.

Participants were required to walk at their comfortable speed (labeled as normal walking (NW)) on a sensorized mat (GAITRite®) for 1 minute. To ensure that steady speed walking was recorded, 2 meters were added at the beginning and at the end of the GAITRite during gait task. Spatiotemporal parameters were analyzed with the ProtoKinetics Movement Analysis Software. Gait assessment protocol is depicted in Figure 1.

The ProtoKinetics software was used for analyzing the spatiotemporal gait parameters. Cadence (number steps × minutes), gait velocity (GV), stride length (SL), and their coefficients of variability (CV) were then determined. Gait parameters were obtained from all steps recorded during the task.

### 2.7. Statistical Analysis

#### 2.7.1. Demographics, Clinical Data, and Gait Assessment

Analyses were performed using Statistical Package for the Social Sciences (SPSS) version 22, and means and standard deviations (SD) were calculated for all dependent variables. Gender differences among groups (PD FOG+, PD FOG-, and HS) were assessed using the chi-square procedure. For age and education, group differences were assessed by the nonparametric Kruskal-Wallis test and group-to-group comparison was performed using the Mann-Whitney *U* test. For gait kinematic parameters that were normally distributed, a one-way ANOVA was used to perform group comparison. For clinical data, the comparison between the PD FOG+ and PD FOG- groups was done using an unpaired *t*-test. *p* values < 0.05 were considered significant.

#### 2.7.2. Functional MRI

One explanatory variable (EV) was defined to model the on-off periods of the task (action observation (AO)) and convolved with the hemodynamic response function (HRF), to detect task-related activity. The 24 motion parameters calculated during motion correction were added as confound EVs together with the mean CSF and WM signals.

A one-sample *t*-test was used to model group mean activation for both PD patients and HS. Differences between the two groups were investigated using a two-sample unpaired *t*-test, adding age and gender as covariates. Moreover, to test for significant differences among the FOG+, FOG-, and HS groups, ANCOVA, with age and gender as covariates, was used.

Results were converted to *Z* values and then thresholded at *Z* = 2.3 for cluster formation and significance threshold corrected for multiple comparisons (*p* < 0.05).

The correlations between brain activations of HS or PD patients (both FOG- and FOG+ groups) and the behavioral measures, in particular GV, SL, and their CV (i.e., GV-CV and SL-CV), were modeled separately with age and gender as covariates. *Z*-maps were thresholded at *Z* ≥ 2.3 for cluster formation, followed with a significance threshold of *p* = 0.05 (cluster corrected using the Gaussian Random Field Theory). Brain functional activations were labeled using the Eickhoff atlas (SPM Anatomy toolbox) [26].

## 3. Results

### 3.1. Demographics and Clinical Data

Two PD subjects could not complete the MRI examination. Moreover, two patients were excluded from the analysis due to gait data corruption. Thus, results were obtained from 50 participants (29 PD and 21 HS). Twelve PD subjects were confirmed to experience FOG, by the new FOG questionnaire [19] and the rapid 360-degree turn in place task [20], whereas the rest of the participants (*n* = 17) were classified as FOG-. At the end of the recruitment phase, age, sex, and education levels were similar among the three groups (PD FOG+, PD FOG-, and HS; *p* always > 0.05). As expected, a significant difference was found for MoCA score among the three groups (*p* < 0.001). Post hoc analysis revealed that the score was significantly lower in both groups of PD participants compared to the HS group (*p* always < 0.001), but similar between PD patients with and without FOG (*p* = 0.131).

For clinical data, statistical analysis did not show a significant difference for disease duration (*p* = 0.633), H&Y stage (*p* = 0.061), MDS-UPDRS motor part (*p* = 0.090), and Levodopa Equivalent Daily Dose (LEDD, *p* = 0.087) between the PD FOG+ and PD FOG- groups. All the details for demographic, clinical characteristics, and statistics are reported in Table 1.

### 3.2. Action Observation (AO) Task-fMRI

#### 3.2.1. Single Group Activations

**Figure 2:**
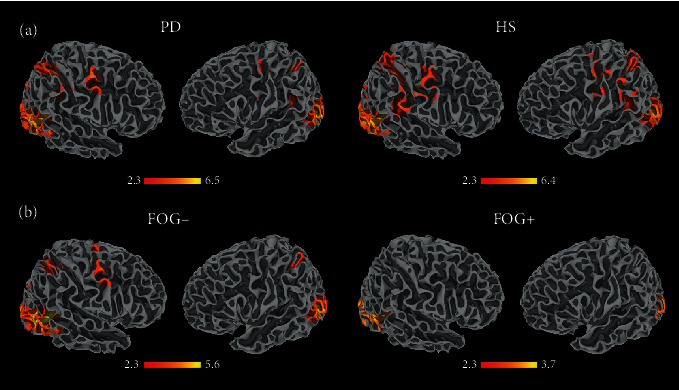
Cortical activity during AO of gait in the (a) whole group of PD patients and in the HS group and in (b) FOG- and FOG+ patients. The results are cluster corrected for multiple comparisons (*Z* ≥ 2.3, *p* < 0.05) and are shown in MNI space.

During the AO task, the whole PD group showed several clusters of activation at the level of the occipital and temporal regions, inferior and superior parietal lobule (IPL and SPL, respectively), and precentral gyrus, in both hemispheres. HS activated at the level of the temporal and occipital regions bilaterally, bilateral SPL and intraparietal sulcus (IPS), left pre- and postcentral gyrus, and superior frontal gyrus (Figure 2(a); Table S1 Supplementary Information). When the FOG- and FOG+ groups were investigated separately, the former showed activity at the level of the right temporal and frontal regions and bilateral occipito-parietal areas, while the latter activated only at the level of the occipital regions (Figure 2(b); Table S2 Supplementary Information).

### 3.3. Subgroup Comparison

#### 3.3.1. PD vs. HS

When the PD and HS groups were compared, HS showed a significantly greater activation at the level of the cingulate cortex, posterior medial frontal cortex (PMFC), occipital regions, and the precuneus (Table S1 Supplementary Information).

#### 3.3.2. PD FOG+ vs. PD FOG- vs. HS (ANCOVA)

**Figure 3:**
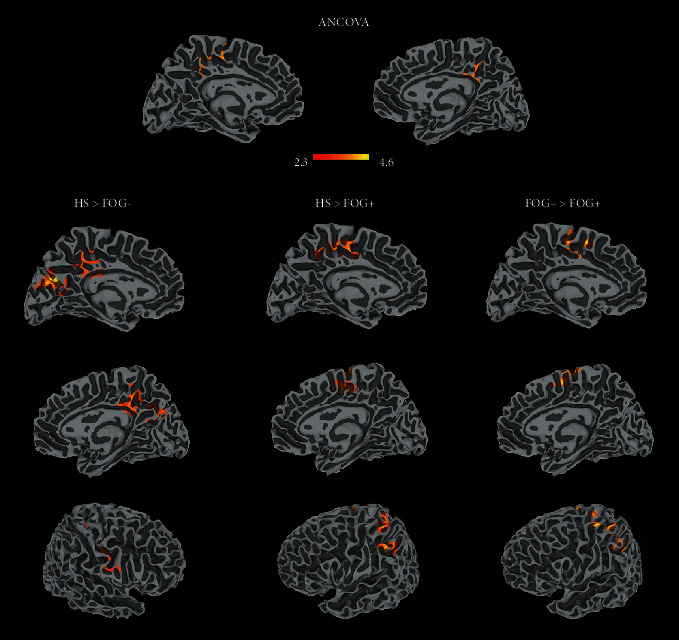
Results of the ANOVA analysis (top) and comparison between subgroups. The results are cluster corrected for multiple comparisons (*Z* ≥ 2.3, *p* < 0.05) and are shown in MNI space.

The analysis revealed a significant difference among the three groups at the level of the left posterior-medial frontal cortex and cingulate cortex. Both FOG- and HS showed an increased activity in the bilateral PMFC, in the left IPL, and in the postcentral gyrus compared to FOG+ participants. Moreover, HS showed two clusters of greater activation, compared to FOG-, at the level of the left occipital regions, right precuneus, left cingulate cortex, and right pre- and postcentral gyrus (Figure 3; Table S3 Supplementary Information).

### 3.4. Gait Performance

As expected, statistical analysis revealed significant differences between the two groups (PD vs. HS) for most of the kinematic parameters obtained during gait task. One-way ANOVA revealed that GV and SL were different among groups (*p* = 0.018 and *p* = 0.012, respectively). Post hoc analysis showed that both FOG+ and FOG- participants had a reduced gait speed (*p* = 0.024 and *p* = 0.012, respectively) and shorter steps (*p* = 0.011 and *p* = 0.014, respectively) compared to HS. Regarding variability, GV-CV and SL-CV were significantly higher (i.e., worse) in patients with PD compared to HS participants (GV-CV *p* = 0.024; SL-CV *p* = 0.003). Post hoc analysis revealed a higher variability in FOG+ and FOG- patients compared to HS (GV-CV: FOG+ vs. HS (*p* = 0.015) and FOG- vs. HS (*p* = 0.032); SL-CV: FOG+ vs. HS (*p* = 0.003) and FOG- vs. HS (*p* = 0.006)). In the FOG+ group, no significant correlation was found between gait parameters and FOG-Q score.

### 3.5. Neuroimaging-Behavioral Correlations

**Figure 4:**
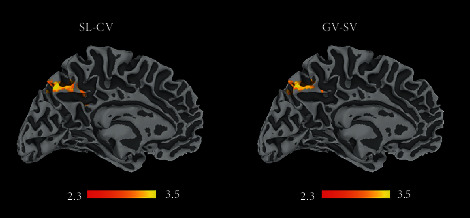
Neuroimaging-behavioral correlations, in particular correlations of brain activity during AO of gait and (a) SL-CV or (b) GV-CV. The results are cluster corrected for multiple comparisons (*Z* ≥ 2.3, *p* < 0.05) and are shown in MNI space.

When brain activations were correlated with kinematic parameters obtained during normal walking task, significant correlations were found in the FOG+ group. Specifically, increased activity at the level of the precuneus cortex was associated with higher SL-CV and GV-CV values (Figure 4, Table S4 Supplementary Information). Statistical analysis did not reveal any significant correlation between cluster significant activations and FOG-Q score or total MDS-UPDRS. Finally, no significant relationships between brain activations and gait parameters were found for the FOG- or HS group.

## 4. Discussion

In this study, we investigated the neural mechanisms underlying AO of gait and the possible association between brain activity and walking performance in PD patients with and without FOG in the on state, under dopaminergic treatment. Three main findings were observed. First, patients with PD present reduced brain activation during AO of normal walking. Second, functional reorganization occurs both in FOG- and FOG+ patients, being more evident in the latter group. Third, in PD FOG+ participants, activity in the precuneus was associated with spatiotemporal parameters of gait.

Although AO is emerging as a tool for rehabilitation of PD symptoms, including FOG, the neural correlates of AO of gait in FOG+ vs. FOG- patients under dopaminergic treatment remain largely unexplored. We showed, in both HS and PD groups, a cortical activation at the level of the occipital, parietal, and frontal areas during the observation of walking. In particular, the pattern of activity we observed in HS is similar to the one reported by Iseki et al. [27]. However, cortical activation was significantly reduced in PD patients, when compared to HS at the level of the PMFC, of occipital areas, and of the precuneus.

In HS, both the occipital areas and the precuneus have been found to be active during observation of gait with precuneus being involved in the spatial control of motor behavior [28]. Both these areas were hypoactivated during real gait in patients with PD, compared to HS [29]. Besides, PMFC is one of the brain areas most consistently associated with gait [27, 29] and found to be affected in patients with PD [28]. Thus, our results confirm the impaired functionality of frontoparietal areas related to gait in patients with PD, even during AO of gait. Finally, previous studies have already shown decreased activity of temporo-occipital regions in PD patients [30, 31] and also in PD with FOG [32] suggesting a possible deficit of visuospatial skills in PD.

When FOG+ and FOG- patients were considered separately, we found that the FOG+ group showed brain activation only at the level of occipital cortex and, compared to both FOG- and HS, presented a reduced activity at the level of association regions such as PMFC and IPL, while the main differences between HS and FOG- was at the level of occipital and primary sensorimotor cortices. Previous studies found a reduced activity in FOG+ patients, compared to FOG- [10], in particular at the level of the supplementary motor area and parietal regions [9] during motor imagery of gait. These results are supported by a recent resting-state functional connectivity study suggesting that FOG might reflect a “widespread increase in intrinsic connectivity within networks in the frontal and parietal areas and basal ganglia as well as a functional disruption between networks implicated in executive and dorsal attention functions” [33]. With this study, we confirm that FOG- and FOG+ differ also in the neural correlates underlying AO of gait and in particular that FOG+ patients present with a more impaired activation.

A previous study on AO in PD patients with FOG [13] revealed a reduced activity in the precentral and SMA areas, in comparison with HS. The preserved activation of PMFC during AO of gait, which we found in FOG- but not in FOG+ patients, adds a missing piece, confirming the involvement of this area in the functional reorganization subtending FOG [34]. Furthermore, the IPL has been described to play a role in the representation of actions triggered by sensory stimuli, including the visual inputs [3, 35]. Therefore, a dysfunction in this area may result in altered sensory input integration and a misrepresentation of the action contributing to the occurrence of FOG. This could be also one of the mechanisms underlying the effectiveness of AO training in improving FOG [12].

Related to the impact of dopaminergic medication on action observation network, a couple of studies analyzed changes in local field potentials recorded from the subthalamic nucleus (STN) during movement observation in on and off conditions [6, 36]. Movement observation was associated with significant changes in the beta oscillatory activity in the STN of PD patients. Particularly, there was a movement observation-related decrease in beta activity in the STN. This decrease, although smaller than those observed during the movement execution, had similar characteristics: it had the same relative amplitude in “on” and “off” and was bilateral and coherent with cortical activity [6]. Differently, the movement-related gamma increase was observed only in the movement execution condition and was modulated by dopaminergic therapy uptake [6]. Overall, these studies confirm what has been suggested related to dopaminergic modulation of network dynamics: dopaminergic medication may induce improvement of basic motor performance by selectively modulating the connectivity in premotor loops at the cortical level as well as cortico-subcortical interactions, but it is not able to efficiently compensate for higher motor control requiring executive functions [37]. In this view, during action observation, different studies suggested the involvement of a complex cortical-subcortical network in order to understand the context, the congruency, and the features of the motor act [38], spreading over association areas that are involved in executive functions. Therefore, the differences observed in the current study could suggest that antiparkinsonian treatment is not sufficient to normalize the neural activity underlying AO in patients with PD, in particular if FOG+ where the impairment of cognitive association areas is more prominent [39]. Lastly, based on the results of Agosta and colleagues [13], it seems the reduced activation of the PMFC in FOG+ compared to controls is not influenced by the antiparkinsonian treatment. Nonetheless, further investigations comparing functional activity in patients in the “on” vs. “off” state during AO of gait are needed to validate these results.

Overall, our results confirm an impaired cortical activation in PD patients, compared to HS, even under the effects of antiparkinsonian medication. Both PD patients with and without FOG undergo a change in the functional connectivity subtending the AO of gait, but a wider modification occurs in PD patients presenting FOG. The knowledge of the pattern of brain activity during AO of gait under the effect of medications could help in understanding the functional modulation shaped by AO training and therefore in designing the most appropriate rehabilitation protocols.

It is worth noting that in our study we did not find significant differences in the spatiotemporal parameters of gait between the two PD groups, even if it is common knowledge that gait features may differ between PD with and without FOG [37, 38]. To elicit FOG episode during a clinical evaluation is not easy. Indeed, in many patients suffering from FOG, despite experiencing FOG during daily living, it is often difficult to observe FOG episodes during examinations conducted in clinical settings. The presence of FOG needs to be often provoked by FOG-provoking tasks or to be measured during more complex circumstances [40–42]. In this study, we measured spatiotemporal parameters of gait during a simple walking task (straight walking at self-selected speed) and this could explain why we did not find differences between the two PD groups. Indeed, it has been previously reported that gait characteristics may be similar during simple gait tasks and differ during complex tasks between FOG+ and FOG- patients [43] mainly because of the nature of the gait task.

Finally, in FOG+ patients, we found a significant association between brain activity during AO of gait and the kinematic parameters recorded during normal walking task. Precisely, the activity at the level of the precuneus correlated with a worse performance in terms of step length and gait variability during normal walking. This association was not significant in PD FOG- patients and in healthy controls. This observation appears to fit with findings in HS where the activation of the precuneus was associated with the imagination of complex locomotor functions such as walking with obstacles [44], attention shift, and the processing of visuospatial stimulus [27]. Therefore, we could speculate that the activity at the level of the precuneus is crucial in the covert action of walking, in those subjects presenting walking difficulties, such as PD patients with FOG [45]. It would be interesting to investigate whether activity in this area might change with AO training.

In this study, there are several limitations we need to acknowledge. First, together with the relatively small sample size of the FOG-/FOG+ subgroups, we did not include a control condition in the “off” state. These are the main reasons for which we propose this article as a pilot investigation. However, this is the first study investigating neural correlates of AO of gait in a population of both FOG+ and FOG- PD patients, revealing that a functional reorganization occurs in PD patients and in particular in those presenting with FOG, which could be linked to a frontoparietal dysfunction. Furthermore, in the latter patients, the behavioral performance during gait is associated with activity at the parietal level (in particular, the precuneus) suggesting that these regions could have a role in AO rehabilitation of gait in FOG+ patients. Second, we did not monitor gaze movements during AO task in the MRI scan. Furthermore, while participants had time to familiarize with the task before the MRI, during the task-fMRI sequence, full adherence to the AO task during the MRI cannot be ensured. Third, gait parameters were recorded during usual walking task and via GAITRite®. Additional studies assessing gait during complex gait circumstances with wearable sensors are calls for better investigating possible relationship between cortical activations and gait features in FOG patients. Finally, further investigations are needed to better elucidate neural changes induced by AOT in PD and to exhibit if differences between patients with and without FOG exist.

## Figures and Tables

**Table 1:** Demographics, clinical characteristics, and gait parameters of participants.

	HS (*n* = 21)	PD FOG- (*n* = 17)	PD FOG+ (*n* = 12)	*p* value
*Demographic and clinical characteristics*				
Age (y, mean ± SD)	64.62 ± 13.52	68.67 ± 4.60	72.00 ± 4.51	*p* = 0.08
Education (y, mean ± SD)	11.16 ± 6.37	9.62 ± 6.44	10.03 ± 4.26	*p* = 0.19
MoCA (score, mean ± SD)	28.81 ± 1.01	26.06 ± 2.79	24.33 ± 2.71	*p* < 0.01
Disease duration (y, mean ± SD)	—	9.24 ± 3.91	10.93 ± 3.54	*p* = 0.633
H&Y (stage, mean ± SD)	—	1.86 ± 0.46	2.20 ± 0.62	*p* = 0.061
MDS-UPDRS III (motor score, mean ± SD)	—	19.11 ± 9.37	26.75 ± 14.09	*p* = 0.090
LEDD	—	558.33 ± 252.99	409.911 ± 197.87	*p* = 0.105
*Gait parameters*				
Gait velocity (cm/s, mean ± SD)	129.95 ± 15.70	113.83 ± 21.41	113.95 ± 20.22	*p* = 0.018
Gait velocity CV (mean ± SD)	3.46 ± 0.91	4.49 ± 1.86	4.77 ± 1.53	*p* = 0.024
Stride length (cm, mean ± SD)	133.96 ± 13.94	122.17 ± 15.32	120.27 ± 13.01	*p* = 0.012
Stride length CV (mean ± SD)	2.20 ± 0.61	3.13 ± 1.06	3.32 ± 1.37	*p* = 0.003
Cadence (*n* steps × min, mean ± SD)	113.94 ± 9.31	110.00 ± 10.89	112.36 ± 15.08	*p* = 0.575

HS: healthy subjects; PD: Parkinson's disease; FOG-: patients without freezing of gait; FOG+: patients with FOG; *n*: number; y: years; SD: standard deviation; MoCA: Montreal Cognitive Assessment; H&Y: Hoehn and Yahr; MDS-UPDRS: Movement Disorder Society-Unified Parkinson Disease Rating Scale; LEDD: Levodopa Equivalent Daily Dose; CV: coefficient of variability.

## Data Availability

The MRI and gait data used to support the findings of this study are available from the corresponding author upon request.

## Supplementary Materials

Supplementary Materials Table S1: clusters of brain activation in PD patients (whole group) and HS and comparison between the two groups. Table S2: clusters of brain activation in FOG- and FOG+ patients. Table S3: subgroup comparison. Table S4: brain behavior correlation results.
