# Supplementary material for: Functional Correlates of Action Observation of Gait in Patients with Parkinson's Disease
Source: Neural Plast. 2020 Dec 29;2020:8869201. doi: 10.1155/2020/8869201 (PMC7787806; doi:10.1155/2020/8869201)
Supplement: Supplementary Materials — Table S1: clusters of brain activation in PD patients (whole group) and HS and comparison between the two groups. Table S2: clusters of brain activation in FOG- and FOG+ patients. Table S3: subgroup comparison. Table S4: brain behavior correlation results. [file 8869201.f1.docx]

**Supplementary Information**

**Action Observation (AO) fMRI task**

*PD vs HS.* When the PD and the HS groups were compared, HS showed a significantly greater activation at the level of the cingulate cortex, posterior medial frontal cortex (PMFC), occipital regions and the precuneus

**Table S1. Clusters of brain activation in PD patients (whole group), HS and comparison between the two groups.**

| **Side** | **Location** | **Cytoarchitetonic location** | **PD patients** | | | | **HS** | | | | **HS>PD** | | | |
| --- | --- | --- | --- | --- | --- | --- | --- | --- | --- | --- | --- | --- | --- | --- |
|  |  |  | **MNI coordinates** | | | **Z score** | **MNI coordinates** | | | **Z score** | **MNI coordinates** | | | **Z score** |
|  |  |  | ***X*** | ***Y*** | ***Z*** |  | ***X*** | ***Y*** | ***Z*** |  | ***X*** | ***Y*** | ***Z*** |  |
| R | **MTG** |  | *46* | *-60* | *0* | 6.35 |  |  |  |  |  |  |  |  |
| R | **ITG** | Area FG2 | *44* | *-64* | *-12* | 5.93 | *44* | *-70* | *-10* | 5.82 |  |  |  |  |
| L | **STG** | Area PFcm | *-48* | *-34* | *22* | 5.1 |  |  |  |  |  |  |  |  |
| R | **IOG** | Area hOc4v | *36* | *-80* | *-8* | 4.73 |  |  |  |  |  |  |  |  |
| R | **IOG** | Area hOc4lp |  |  |  |  | *36* | *-84* | *-4* | 5.75 |  |  |  |  |
| L | **IOG** | Area hOc4lp |  |  |  |  | *-36* | *-96* | *-4* | 5.26 |  |  |  |  |
| L | **IOG** | Area hOc5 | *-44* | *-72* | *-6* | 4.94 |  |  |  |  |  |  |  |  |
| R | **MOG/V5** | Area hOc5 |  |  |  |  | *46* | *-68* | *4* | 6.41 |  |  |  |  |
| L | **MOG/V5** | Area hOc5 | *-44* | *-74* | *2* | 6.57 |  |  |  |  |  |  |  |  |
| R | **MOG** | Area hOc3d | *30* | *-92* | *12* | 4.95 | *30* | *-98* | *12* | 5.19 |  |  |  |  |
| L | **MOG** | Area hOc4la | *-42* | *-86* | *0* | 5.5 | *-46* | *-76* | *2* | 5.38 |  |  |  |  |
| L | **MOG** | Area hOc4d | *-22* | *-90* | *10* | 4.21 |  |  |  |  |  |  |  |  |
| L | **MOG** | Area hOc4lp | *-44* | *-86* | *16* | 4.06 | *-40* | *-88* | *-6* | 4.58 |  |  |  |  |
| L | **MOG** | Area hOc3d |  |  |  |  | *-22* | *-98* | *0* | 5.02 |  |  |  |  |
| L | **SOG** |  |  |  |  |  |  |  |  |  | -14 | -72 | 28 | 4.41 |
| L | **FG** | Area FG2 | *-40* | *-72* | *-18* | 4.09 |  |  |  |  |  |  |  |  |
| L | **CG** |  |  |  |  |  |  |  |  |  | *-16* | *-66* | *14* | 3.95 |
| R | **Prec** |  |  |  |  |  |  |  |  |  | *12* | *-68* | *30* | 4.16 |
| L | **Prec** |  |  |  |  |  |  |  |  |  | *-4* | *-46* | *28* | 4.66 |
| R | **IPL** | Area 2 | *40* | *-38* | *54* | 4.79 | *32* | *-42* | *46* | 3.58 |  |  |  |  |
| R | **IPL** | Area hIP3 | *38* | *-44* | *54* | 4.79 | *36* | *-44* | *52* | 4.16 |  |  |  |  |
| R | **SPL** | Area 7A | *32* | *-56* | *58* | 4.1 |  |  |  |  |  |  |  |  |
| R | **SPL** | Area 7PC |  |  |  |  | *36* | *-46* | *58* | 4.13 |  |  |  |  |
| R | **SPL** | Area 5L |  |  |  |  | *16* | *-56* | *74* | 3.65 |  |  |  |  |
| L | **SPL** | Area hIP3 | *-22* | *-60* | *52* | 3.61 | *-40* | *-50* | *60* | 4.17 |  |  |  |  |
| L | **SPL** | Area 7A | *-26* | *-68* | *64* | 2.8 | *-24* | *-64* | *62* | 3.46 |  |  |  |  |
| L | **SPL** | Area 5L |  |  |  |  | *-18* | *-56* | *72* | 3.34 |  |  |  |  |
| R | **SMG** | Area 2 | *32* | *-40* | *46* | 3.55 |  |  |  |  |  |  |  |  |
| R | **SMG** | Area PF |  |  |  |  | *64* | *-26* | *28* | 4.31 |  |  |  |  |
| L | **SMG** |  | *-50* | *-40* | *28* | 4.21 |  |  |  |  |  |  |  |  |
| R | **MFG** |  | *48* | *2* | *52* | 4.82 |  |  |  |  |  |  |  |  |
| R | **MCC** | Area 5Ci |  |  |  |  |  |  |  |  | *6* | *-38* | *46* | 3.54 |
| L | **MCC** | Area 5M |  |  |  |  |  |  |  |  | *-4* | *-36* | *50* | 3.97 |
| R | **PMFC** |  |  |  |  |  |  |  |  |  | *10* | *-8* | *50* | 3.34 |
| R | **PrecG** | Area 44 | *56* | *4* | *40* | 3.4 |  |  |  |  |  |  |  |  |
| L | **PrecG** |  | *-44* | *-8* | *54* | 3.24 | *-52* | *-6* | *52* | 4.54 |  |  |  |  |
| L | **PostG** |  | *-52* | *-8* | *54* | 3.44 | *-62* | *-6* | *36* | 3.38 |  |  |  |  |
| L | **SFG** |  |  |  |  |  | *-34* | *-4* | *66* | 3.41 |  |  |  |  |
| Abbreviations: R: right; L: left; MTG: middle temporal gyrus; ITG: inferior temporal gyrus; STG: superior temporal gyrus; IOG: inferior occipital gyrus; MOG: middle occipital gyrus; SOG: superior occipital gyrus; CG: calcarine gyrus; FG: fusiform gyrus; IPL: inferior parietal lobule; SPL: superior parietal lobule; SMG: supramarginal gyrus; Prec: precuneus; MCC: mid-cingulate cortex; PMFC: postero-medial frontal cortex; MFG: middle frontal gyrus; SFG: superior frontal gyrus; PrecG: precentral gyrus; PostG: postcentral gyrus. | | | | | | | | | | | | | | |

**Table S2. Clusters of brain activation in FOG- and FOG+ patients.**

| **Side** | **Location** | **Cytoarchitetonic location** | **FOG-** | | | | **FOG+** | | | |
| --- | --- | --- | --- | --- | --- | --- | --- | --- | --- | --- |
|  |  |  | **MNI coordinates** | | | **Z score** | **MNI coordinates** | | | **Z score** |
|  |  |  | ***X*** | ***Y*** | ***Z*** |  | ***X*** | ***Y*** | ***Z*** |  |
| R | **MTG** | Area hOc4la |  |  |  |  | *54* | *-70* | *6* | 3.25 |
| R | **ITG** |  | *46* | *-54* | *-6* | 4.88 |  |  |  |  |
| R | **ITG** | Area FG2 | *44* | *-66* | *-10* | 4.71 |  |  |  |  |
| R | **MOG/V5** | Area hOc5 | *48* | *-64* | *2* | 5.66 |  |  |  |  |
| R | **MOG** | Area hOc4lp |  |  |  |  | *42* | *-90* | *4* | 3.45 |
| L | **MOG** | Area hOc4lp |  |  |  |  | *-34* | *-86* | *-2* | 3.23 |
| R | **MOG** | Area hO4la | *44* | *-76* | *4* | 5.42 |  |  |  |  |
| L | **MOG** | Area hO4la | *-42* | *-78* | *4* | 5.24 | *-44* | *-72* | *4* | 3.44 |
| L | **MOG** | Area hOc4la |  |  |  |  | *-44* | *-72* | *4* | 3.44 |
| L | **MOG** | Area hO4lp | *-42* | *-86* | *2* | 4.71 |  |  |  |  |
| R | **MOG** | Area hOc3d | *28* | *-96* | *8* | 4.71 |  |  |  |  |
| L | **MOG** | Area hOc5 | *-42* | *-74* | *4* | 5.32 |  |  |  |  |
| R | **SOG** | Area hOc2 |  |  |  |  | *26* | *-100* | *10* | 3.24 |
| R | **LG** | Area hOc3v |  |  |  |  | *20* | *-84* | *-10* | 3.19 |
| R | **IPL** | Area hIP3 | *36* | *-46* | *54* | 4.69 |  |  |  |  |
| R | **SMG** | Area 2 | *32* | *-40* | *46* | 3.6 |  |  |  |  |
| R | **SPL** | Area 7PC | *36* | *-54* | *64* | 3.42 |  |  |  |  |
| R | **SPL** | Area 7A | *22* | *-62* | *52* | 2.96 |  |  |  |  |
| L | **IPL** |  | *-32* | *-44* | *48* | 3.94 |  |  |  |  |
| L | **IPL** | Area hIP3 | *-30* | *-56* | *54* | 3.72 |  |  |  |  |
| L | **SPL** | Area 7PC | *-34* | *-52* | *64* | 3.27 |  |  |  |  |
| R | **PMFC** |  | *8* | *-2* | *66* | 3.75 |  |  |  |  |
| R | **SFG** |  | *26* | *-2* | *64* | 3.71 |  |  |  |  |
| R | **MFG** |  | *52* | *-2* | *54* | 4.69 |  |  |  |  |
| R | **PrecG** |  | *52* | *2* | *50* | 3.72 |  |  |  |  |
| Abbreviations: R: right; L: left; MTG: middle temporal gyrus; ITG: inferior temporal gyrus; MOG: middle occipital gyrus; SOG: superior occipital gyrus; LG: lingual gyrus; IPL: inferior parietal lobule; SPL: superior parietal lobule; SMG: supramarginal gyrus; PMFC: postero-medial frontal cortex; MFG: middle frontal gyrus; SFG: superior frontal gyrus; PrecG: precentral gyrus. | | | | | | | | | | |

**Table S3. Subgroups comparison**

| **Side** | **Location** | **Cytoarchitetonic location** | **FOG- > FOG+** | | | | **HS > FOG-** | | | | **HS > FOG+** | | | |
| --- | --- | --- | --- | --- | --- | --- | --- | --- | --- | --- | --- | --- | --- | --- |
|  |  |  | **MNI coordinates** | | | **Z score** | **MNI coordinates** | | | **Z score** | **MNI coordinates** | | | **Z score** |
|  |  |  | ***X*** | ***Y*** | ***Z*** |  | ***X*** | ***Y*** | ***Z*** |  | ***X*** | ***Y*** | ***Z*** |  |
| **L** | **SOG** |  |  |  |  |  | *-14* | *-72* | *28* | 4.69 |  |  |  |  |
| **L** | **Cuneus** |  |  |  |  |  | *-4* | *-76* | *28* | 4.56 |  |  |  |  |
| **L** | **CG** |  |  |  |  |  | *-16* | *-66* | *16* | 4 |  |  |  |  |
| R | **Prec** |  |  |  |  |  | *12* | *-68* | *30* | 3.99 |  |  |  |  |
| L | **IPL** | Area PFt | *-54* | *-32* | *42* | 3.48 |  |  |  |  | *-56* | *-30* | *42* | 3.7 |
| L | **IPL** | Area PF |  |  |  |  |  |  |  |  | *-54* | *-40* | *44* | 3.28 |
| L | **IPL** | Area hIP2 | *-50* | *-46* | *38* | 3.35 |  |  |  |  |  |  |  |  |
| L | **IPL** | Area hIP1 | *-46* | *-50* | *44* | 3.31 |  |  |  |  |  |  |  |  |
| L | **MCC** |  |  |  |  |  |  |  |  |  | *-8* | *-22* | *40* | 3.47 |
| L | **ParaG** | Area 5M |  |  |  |  | *-4* | *-38* | *50* | 3.69 | *-4* | *-32* | *50* | 3.4 |
| R | **PMFC** |  | *6* | *-12* | *68* | 3.92 |  |  |  |  | *6* | *-12* | *68* | 3.45 |
| L | **PMFC** |  | *-2* | *4* | *54* | 3.7 |  |  |  |  | *-4* | *-18* | *50* | 4.28 |
| R | **PrecG** |  |  |  |  |  | *50* | *-12* | *46* | 3.14 |  |  |  |  |
| L | **PrecG** |  | *-44* | *-16* | *60* | 3.72 |  |  |  |  |  |  |  |  |
| R | **PostG** | Area 3a |  |  |  |  | *44* | *-12* | *32* | 3.16 |  |  |  |  |
| R | **PostG** | Area4p |  |  |  |  | *50* | *-12* | *32* | 3.08 |  |  |  |  |
| L | **PostG** | Area 1 | *-46* | *-30* | *60* | 3.42 |  |  |  |  | *-34* | *-40* | *64* | 3.75 |
| Abbreviations: R: right; L: left; SOG: superior occipital gyrus; CG: calcarine gyrus; Prec: precuneus; IPL: inferior parietal lobule; MCC: mid-cingulate cortex; PMFC: postero-medial frontal cortex; MFG: middle frontal gyrus; PrecG: precentral gyrus; PostG: postcentral gyrus; ParaG: paracentral gyrus. | | | | | | | | | | | | | | |

**Table S4. Brain behavior correlations results**

| **Side** | **Location** | **Cytoarchitectonic location** | **MNI coordinates** | | | **Z score** |
| --- | --- | --- | --- | --- | --- | --- |
|  |  |  | *X* | *Y* | *Z* |  |
| **GV_CV during normal walking** | | | | | | |
| L | **Prec** | Area 7P | *-2* | *-78* | *50* | 3.46 |
| L | **SOG** |  | *-14* | *-78* | *44* | 3.18 |
| L | **Prec** | Area 7A | *-6* | *-64* | *48* | 3.13 |
| **SL_CV during normal walking** | | | | | | |
| L | **Prec** | Area 7P | *-2* | *-78* | *50* | 3.52 |
| L | **Prec** | Area 7A | *-6* | *-64* | *48* | 3.14 |
| L | **SOG** |  | *-14* | *-78* | *44* | 3.07 |
| Abbreviations: R: right; L: left; Prec: precuneus; SOG: superior occipital gyrus. | | | | | | |
